# The association between albumin-corrected calcium and prognosis in patients with cardiac arrest: a retrospective study based on the MIMIC-IV database

**DOI:** 10.1186/s40001-024-01841-4

**Published:** 2024-04-24

**Authors:** Lei Zhong, Jianhong Lu, Xu Sun, Yuechen Sun

**Affiliations:** 1 Department of Intensive Care Unit, Huzhou Central Hospital, Affiliated Central Hospital Huzhou University, Huzhou, 313000 China; 2 Department of General Surgery, Huzhou Central Hospital, Affiliated Central Hospital Huzhou University, Huzhou, 313000 China; 3 Department of Emergency, Huzhou Central Hospital, Affiliated Central Hospital Huzhou University, Huzhou, 313000 China; 4 Present Address: The Fifth School of Clinical Medicine of Zhejiang Chinese Medical University, Huzhou, 313000 China

**Keywords:** Albumin-corrected calcium, Cardiac arrest, Prognosis

## Abstract

**Background:**

Cardiac arrest (CA) is one of the leading causes of death globally, characterized by high incidence and mortality. It is of particular significance to determine the prognosis of patients with CA early and accurately. Therefore, we aim to investigate the correlation between albumin-corrected calcium (ACC) and the prognosis in patients diagnosed with CA.

**Methods:**

We retrospectively collected data from medical information mart for intensive care IV database. Patients were divided into two groups (survival and non-survival groups), according to the 90-day prognosis. In the Restricted cubic spline (RCS) analysis, the cut-off values (8.86 and 10.32) were obtained to categorize patients into three groups: low ACC group (< 8.86), moderate ACC group (8.86–10.32), and high ACC group (> 10.32). The least absolute shrinkage and selection operator with a ten-fold cross-validation regression analysis was performed to identify variables linked to the mortality. The inverse probability treatment weighting (IPTW) was used to address the confounding factors, and a weighted cohort was generated. RCS, Kaplan–Meier curve, and Cox regression analyses were used to explore the relationship between ACC and the mortality. Sensitivity analysis was employed to validate the stability of the results.

**Results:**

Cut-off values for ACC of 8.86 and 10.32 were determined. RCS analyses showed that there was an overall non-linear trend relationship between ACC and the risk of 90-day and 360-day mortalities. After IPTW adjustment, compared to the moderate ACC group, the 90-day and 360-day mortalities in the high ACC group were higher (*P* < 0.05). The Cox analyses before and after IPTW adjustment showed that both low ACC and high ACC group were independent risk factors for 90-day and 360-day all-cause mortality in patients with CA (*P* < 0.05). The results obtained from sensitivity analyses indicated the stability of the findings. The Kaplan–Meier survival curves indicated that 90- and 360-day cumulative survival rates in the low ACC and high ACC groups were lower than that in the moderate ACC group (χ^2^ = 11.350, *P* = 0.003; χ^2^ = 14.110,* P* = 0.001).

**Conclusion:**

Both low ACC (< 8.86) and high ACC groups (> 10.32) were independent risk factors for 90-day and 360-day all-cause mortality in patients with CA (*P* < 0.05). For those CA patients with high and low ACC, it deserved the attention of clinicians.

**Supplementary Information:**

The online version contains supplementary material available at 10.1186/s40001-024-01841-4.

## Introduction

Cardiac arrest (CA), characterized by high incidence and mortality, is one of the leading causes of death globally. There are over 340,000 cases of out-of-hospital cardiac arrest (CA) annually, with an estimated occurrence of around 292,000 in-hospital CA events per year [1]. In China, there are over 230 million individuals with cardiovascular disease, and 550,000 people experience CA annually [2]. Despite significant resources and efforts being dedicated to improving CA patient outcomes, the overall survival rate of out-of-hospital CA patients upon hospital discharge remains below 10% [3, 4]. Therefore, it is of particular significance to determine the prognosis of patients with CA early and accurately. While numerous indicators like neuron-specific enolase, anion gap, fibroblast growth factor 23, albumin-corrected anion gap, etc., have been utilized to predict prognosis [5–8], the diagnostic value remains unconfirmed, with only neuron-specific enolase being endorsed by the 2006 American Neurological Association guidelines [6]. This underscores the ongoing urgency in identifying simple yet effective prognostic indicators for patients with CA.

Albumin-corrected calcium (ACC) is used to assess ionized calcium levels. It is obtained by adjusting the total calcium concentration based on the serum albumin level, taking into account of the influence of albumin on calcium ions. For this reason, clinicians mostly use ACC to assess free calcium levels [9]. Researches have shown that low calcium is an independent risk factor for mortality in patients suffering from pulmonary embolism and acute myocardial infarction (AMI) [10, 11]. However, there is limited research on the harm caused by high calcium levels. Therefore, this study aimed to explore the association between different ACC levels and prognosis of CA patients.

## Materials and methods

### Study design

We conducted a retrospective cohort study and collected data from all eligible adult patients in the Medical Information Mart for Intensive Care IV database (MIMIC-IV, v2.0, 2008–2019) [12]. The collection of patient information and creation of the research resource was reviewed by the Institutional Review Board at the Beth Israel Deaconess Medical Center, who granted a waiver of informed consent and approved the data sharing initiative, and the authors of the study were granted access to the database (ID number: 42303155, 53446653). Our study adhered to the STROBE statement guidelines which was shown in Additional file 1.

### Population and data extraction

The inclusion criteria of the study were as follows: (1) Patients with CA aged 18 years or older. (2) Patients admitted to the intensive care unit (ICU) for the first time. The following exclusion criteria were applied: (1) ICU stays shorter than 24 h. (2) Variables such as albumin and serum calcium were not measured on the day of admission to the ICU.

Clinic data of the patients included age, sex, sequential organ failure assessment (SOFA) score, serum calcium, albumin, ACC, white blood cell (WBC) count, hemoglobin, platelet count, red blood cell distribution width (RDW), mean corpuscular volume (MCV), anion gap, blood urea nitrogen (BUN), creatinine, alanine aminotransferase (ALT), aspartate aminotransferase (AST), glucose, bilirubin, serum potassium, serum phosphate, the number of patients who underwent transthoracic echocardiography, norepinephrine and dobutamine administration, mechanical ventilation (MV), continuous renal replacement therapy, intra-aortic balloon pump (IABP), and defibrillation. Also, comorbidities and ICU length of stay (LOS) were collected. Comorbidities included were as follows: hypertension, diabetes, acute kidney injury (AKI), chronic pulmonary disease, deep vein thrombosis (DVT), ventricular fibrillation (VF), congestive heart failure, cerebrovascular disease, malignant tumor, AMI, and cardiogenic shock. All the results were data from the first test conducted after ICU admission.

Based on the previous literature [9], the calculation formula for ACC was as follows: ACC = serum total calcium (mg/dL) + 0.8 * [4.0 – serum albumin (g/dL)].

### Groups and outcomes

In terms of the 90-day prognosis, patients were divided into survival group (*n* = 302) and non-survival group (*n* = 496).

In the restricted cubic spline (RCS) analysis, the cut-off values (8.86 and 10.32) at which the hazard ratio (HR) for 90-day mortality risk equals 1 were used to categorize patients into three groups: the low ACC group (< 8.86, *n* = 399), the moderate ACC group (8.86–10.32, *n* = 328), and the high ACC group (> 10.32, *n* = 71).

The main focus of this study was the 90-day all-cause mortality, considered as the primary endpoint, while the 360-day all-cause mortality served as the secondary endpoint.

### Statistical analysis

For continuous data that followed a normal distribution, it was presented as mean ± standard deviation ($\overline{{\text{x}} }$ ± s), and the t-test was used for between-group comparisons. If the data did not adhere to a normal distribution, it was presented as median (interquartile range) [M (QL, QU)], and the Mann–Whitney U test was used for between-group comparisons. Discrete data were presented as composition ratios (%), and the chi-square (χ^2^) test method was applied for between-group comparisons.

After adjusting for confounding factors, RCS analyses were used to examine the relationship between ACC and the risk of mortality, ultimately obtaining cut-off values for grouping. The least absolute shrinkage and selection operator (LASSO) with a tenfold cross-validation regression analysis was performed on variables aiming to further identify variables linked to the 90-day and 360-day prognosis. Variables selected through LASSO analysis were included in the Cox regression analyses to examine the relationship between ACC and the 90-day and 360-day mortalities. In order to address confounding factors and weighted samples, we employed the inverse probability treatment weighting (IPTW) method to construct the weighted cohort using the original population. Variables with a final *P* < 0.10 were included in another multivariable Cox regression. The results were presented as HR and 95% confidence intervals (CI). Kaplan–Meier curves before and after IPTW adjustment were created and log-rank tests were utilized to compare the cumulative survival rates between the three groups with different ACC levels. Sensitivity analysis was employed to validate the stability of the results.

Stata14.0 and R4.2.3 software were used for data analysis, and *P* < 0.05 was considered statistically significant.

## Results

### Participants and characteristic

**Fig. 1:**
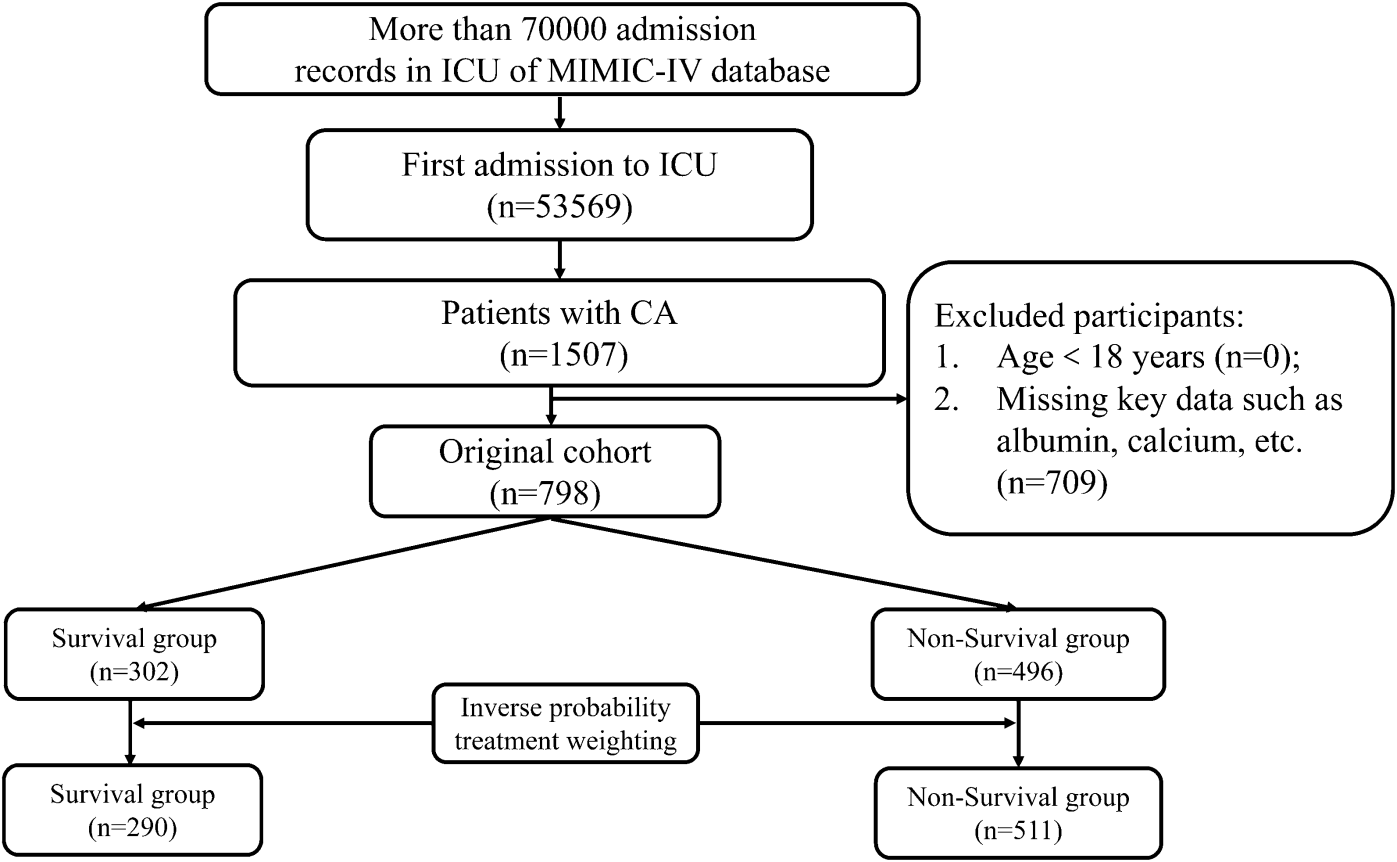
Participants selection process

Ultimately, a total of 798 eligible patients, as shown in Fig. 1, were included with an average age of 65.00 ± 17.45 years in this study. After the analysis of IPTW, a weighted cohort was generated with a total population of 801.37, where the number of individuals in survival group was 290, and in non-survival group, it was 511. Age, SOFA score, ACC, RDW, MCV, anion gap, BUN, creatinine, ALT, AST, serum phosphate, comorbidities (cerebrovascular disease, malignant tumor), and the proportion of receiving transthoracic echocardiography and norepinephrine in the non-survival group were higher (*P* < 0.05). Albumin, hemoglobin, ICU LOS, the proportion of VF, and congestive heart failure in the survival group were higher (*P* < 0.05). In the weighted cohort, age, sex, SOFA score, albumin, ACC, hemoglobin, RDW, MCV, AST, BUN, creatinine, anion gap, phosphate, comorbidities including congestive heart failure, VF, and malignant tumor, the proportion of receiving norepinephrine, and ICU LOS between survival and non-survival groups were statistically significant (*P* < 0.05), as shown in Table 1.

**Table 1:** Baseline characteristics of the two groups

Variables	Original cohort			Weighted cohort		
	Survival group (*n* = 302)	Non-survival group (*n* = 496)	*P*-value	Survival group (*n* = 290)	Non-survival group (*n* = 511)	*P*-value
Age (year)	62.52 ± 16.59	66.51 ± 17.80	0.002	62.89 ± 16.14	68.06 ± 17.67	< 0.001
Sex, male, *n* (%)	196 (64.90)	304 (61.29)	0.307	181.20 (62.50)	323.30 (63.20)	< 0.001
SOFA score	9.00 (5.00, 12.00)	11.00 (8.00, 14.00)	< 0.001	9.00 (5.00, 12.00)	10.72 (7.00, 13.00)	< 0.001
Serum calcium (mg/L)	8.33 ± 1.08	8.28 ± 1.60	0.643	8.31 ± 1.06	8.22 ± 1.50	0.393
Albumin (g/dL)	3.40 ± 0.70	3.04 ± 0.75	< 0.001	3.37 ± 0.71	3.05 ± 0.72	< 0.001
ACC (mg/dl)	8.81 ± 1.01	9.05 ± 1.63	0.021	8.81 ± 0.96	8.98 ± 1.50	0.066
WBC (× 10^9^/L)	12.20 (8.60, 17.40)	13.00 (8.65, 18.90)	0.309	12.20 (8.52, 17.00)	13.30 (9.20, 19.80)	0.086
Hemoglobin (g/L)	121.18 ± 27.46	115.16 ± 27.94	0.003	119.58 ± 30.15	113.27 ± 28.04	0.042
Platelet (× 10^9^/L)	209.50 (158.00, 269.00)	200.50 (139.50, 274.00)	0.348	209.00 (158.00, 267.00)	205.00 (142.00, 286.72)	0.895
RDW (%)	14.61 ± 2.25	15.34 ± 2.44	< 0.001	14.75 ± 2.44	15.53 ± 2.46	0.005
MCV (fL)	92.60 ± 7.53	94.29 ± 8.35	0.004	92.67 ± 7.70	94.54 ± 8.39	0.010
Anion gap (mmol/L)	17.65 ± 5.29	20.09 ± 6.43	< 0.001	17.58 ± 5.37	20.23 ± 6.81	< 0.001
BUN (mg/dL)	7.48 (4.98, 11.39)	9.61 (6.05, 16.38)	< 0.001	7.48 (5.28, 11.75)	9.97 (6.41, 17.44)	< 0.001
creatinine (umol/L)	106.08 (79.56, 150.28)	132.60 (88.40, 194.48)	< 0.001	106.08 (79.56, 159.12)	132.60 (97.24, 203.32)	0.001
ALT (U/L)	53.00 (27.00, 139.00)	72.50 (29.00, 219.50)	0.004	52.00 (26.00, 140.89)	63.00 (29.00, 183.98)	0.114
AST (U/L)	81.00 (40.00, 191.00)	119.50 (47.50, 373.00)	< 0.001	81.00 (39.00, 195.66)	102.00 (41.00, 322.10)	0.038
Glucose (mmol/L)	9.11 (6.89, 13.00)	9.47 (6.97, 14.22)	0.356	9.28 (7.17, 13.05)	9.00 (6.65, 13.85)	0.697
Bilirubin (umol/L)	10.26 (6.84, 15.39)	10.26 (6.84, 18.81)	0.312	10.26 (6.84, 15.39)	10.26 (6.84, 18.81)	0.291
Potassium (mmol/L)	4.49 ± 1.18	4.56 ± 1.09	0.436	4.50 ± 1.16	4.56 ± 1.09	0.553
Phosphate (mmol/L)	1.43 ± 0.65	1.70 ± 0.82	< 0.001	1.46 ± 0.67	1.69 ± 0.83	0.002
Transthoracic echocardiography, *n* (%)	128 (42.38)	169 (34.07)	0.018	114.10 (39.30)	175.20 (34.30)	0.200
Norepinephrine, *n* (%)	170 (56.29)	355 (71.57)	< 0.001	162.70 (56.10)	350.10 (68.50)	0.005
Dobutamine, *n* (%)	15 (4.97)	35 (7.06)	0.237	15.80 (5.50)	32.20 (6.30)	0.651
MV, *n* (%)	259 (85.76)	441 (88.91)	0.189	248.40 (85.60)	437.50 (85.60)	0.986
CRRT, *n* (%)	26 (8.61)	58 (11.69)	0.169	30.0 (10.30)	53.8 (10.50)	0.956
IABP, *n* (%)	16 (5.30)	23 (4.64)	0.674	17.50 (6.00)	22.40 (4.40)	0.337
Defibrillation, *n* (%)	17 (5.63)	21 (4.23)	0.369	17.10 (5.90)	22.40 (4.40)	0.380
Comorbidities, *n* (%)						
Hypertension	119 (39.40)	184 (37.10)	0.515	111.10 (38.30)	187.90 (36.70)	0.693
Diabetes	93 (30.79)	152 (30.65)	0.965	100.90 (34.80)	155.20 (30.40)	0.268
AKI	246 (81.46)	411 (82.86)	0.614	239.90 (82.70)	431.30 (84.4)	0.555
Chronic pulmonary disease	80 (26.49)	120 (24.19)	0.468	76.30 (26.30)	119.70 (23.40)	0.408
Pulmonary embolism	18 (5.96)	22 (4.44)	0.338	16.20 (5.60)	27.10 (5.30)	0.874
DVT	11 (3.64)	19 (3.83)	0.892	13.10 (4.50)	26.00 (5.10)	0.772
VF	70 (23.18)	69 (13.91)	0.001	65.10 (22.40)	67.40 (13.20)	0.002
Congestive heart failure	134 (44.37)	165 (33.27)	0.002	131.90 (45.50)	186.00 (36.40)	0.037
Cerebrovascular disease	32 (10.60)	84 (16.94)	0.014	31.40 (10.80)	82.80 (16.20)	0.051
Malignant tumor	26 (8.61)	68 (13.71)	0.030	23.20 (8.00)	79.50 (15.50)	0.014
AMI	69 (22.85)	103 (20.77)	0.488	65.20 (22.50)	101.10 (19.80)	0.398
Cardiogenic shock	55 (18.21)	88 (17.74)	0.867	52.10 (18.00)	92.90 (18.20)	0.949
ICU LOS (d)	4.55 (2.29, 8.90)	2.66 (1.06, 6.32)	< 0.001	4.55 (2.32, 8.69)	2.65 (1.07, 6.15)	< 0.001

ACC: albumin-corrected calcium; AKI: acute kidney injury; ALT: alanine aminotransferase; AMI: acute myocardial infarction; AST: aspartate aminotransferase; BUN: blood urea nitrogen; CRRT: continuous renal replacement therapy; DVT: deep vein thrombosis; IABP: intra-aortic balloon pump; ICU: intensive care unit; LOS: length of stay; MCV: mean corpuscular volume; MV: mechanical ventilation; RDW: red blood cell distribution width; SOFA: sequential organ failure estimate; VF: ventricular fibrillation; WBC: white blood cell

### Cut-off values and RCS analyses

Cut-off values for ACC of 8.86 and 10.32 were determined based on ACC values at a 90-day mortality hazard ratio (HR) of 1 in the RCS analysis. Subsequently, patients were categorized into low ACC (< 8.86, *n* = 399), moderate ACC (8.86–10.32, *n* = 328), and high ACC (> 10.32, *n* = 71) groups.

**Fig. 2:**
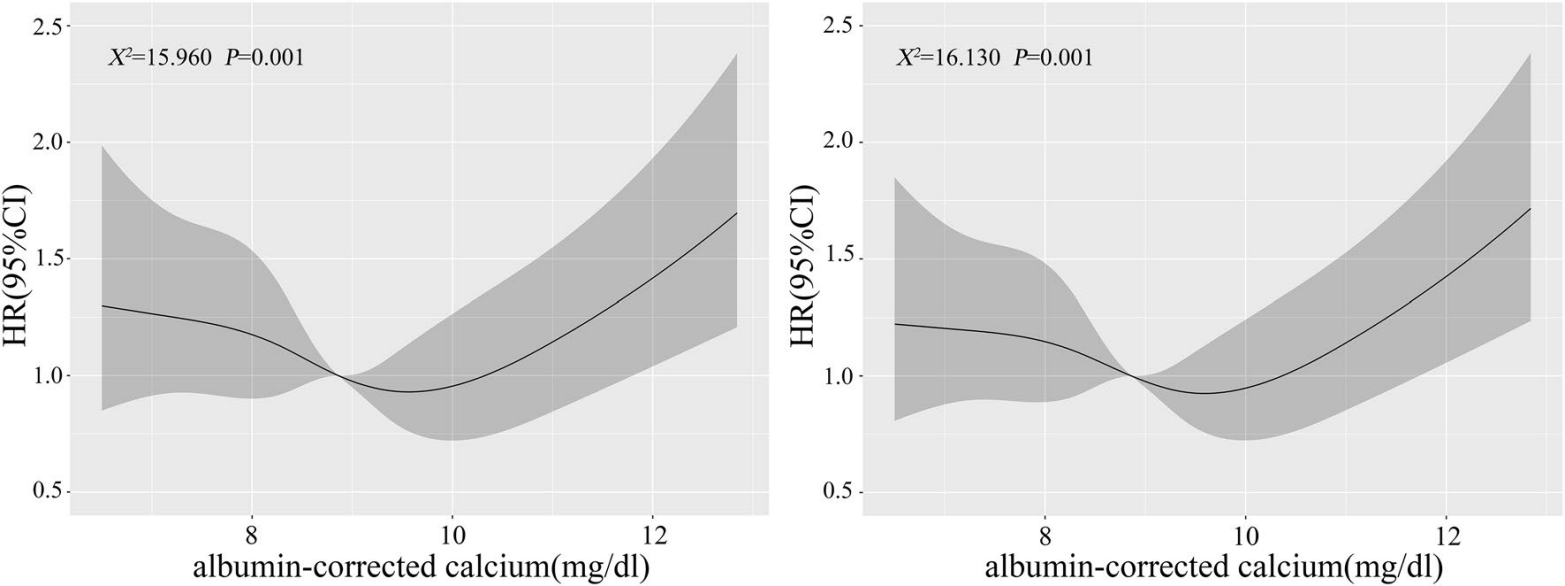
RCS analyses of the correlation between albumin-corrected calcium (ACC) and the risk of 90-day (left) and 360-day (right) all-cause mortality in patients with cardiac arrest (CA)

After adjusting for the 17 variables with *P* < 0.05 from Table 1, RCS analyses in Fig. 2 showed that there was an overall non-linear trend relationship between ACC and the risk of 90-day and 360-day all-cause mortality. Simultaneously, we observed that when ACC value was less than 8.86 or greater than 10.32, the 90-day all-cause mortality risk of patients shows an upward trend. A similar trend was also observed between ACC and the 360-day all-cause mortality risk of patients with CA (χ^2^ = 15.960, *P* = 0.001; χ^2^ = 16.130, *P* = 0.001).

### ACC and all-cause mortality

As shown in Table 2, the 90-day and 360-day all-cause mortalities of the patients with CA were 62.16% and 67.42%, respectively. There existed significant differences in overall mortality among the three groups (*P* < 0.05). However, upon further comparison, the 90-day and 360-day mortalities did not show a statistically significant difference between the moderate and low ACC groups (*P* > 0.05). Compared to the moderate ACC group, the 90-day and 360-day mortalities in the high ACC group were higher (*P* < 0.05).

**Table 2:** **90**-day and 360-day all-cause mortality for three groups of patients with CA

Group	90-day		χ^2^	*P-*value	360-day		χ^2^	*P-*value
	Survival (*n* = 302)	Non-survival (*n* = 496)			Survival (*n* = 260)	Non-survival (*n* = 538)		
< 8.86 (*n* = 399)	146 (36.59)	253 (63.41)*			131 (32.83)	268 (67.17)*		
8.86–10.32 (*n* = 328)	139 (42.38)	189 (57.62)			118 (35.98)	210 (64.02)		
> 10.32 (*n* = 71)	17 (23.94)	54 (76.06)^#^	8.965	0.011	11 (15.49)	60 (84.51)^#^	11.170	0.004

*Compared to the moderate ACC group, there was no statistically significant difference (*P* > 0.05)

^#^Compared to the moderate ACC group, the mortality was higher (*P* < 0.05)

**Fig. 3:**
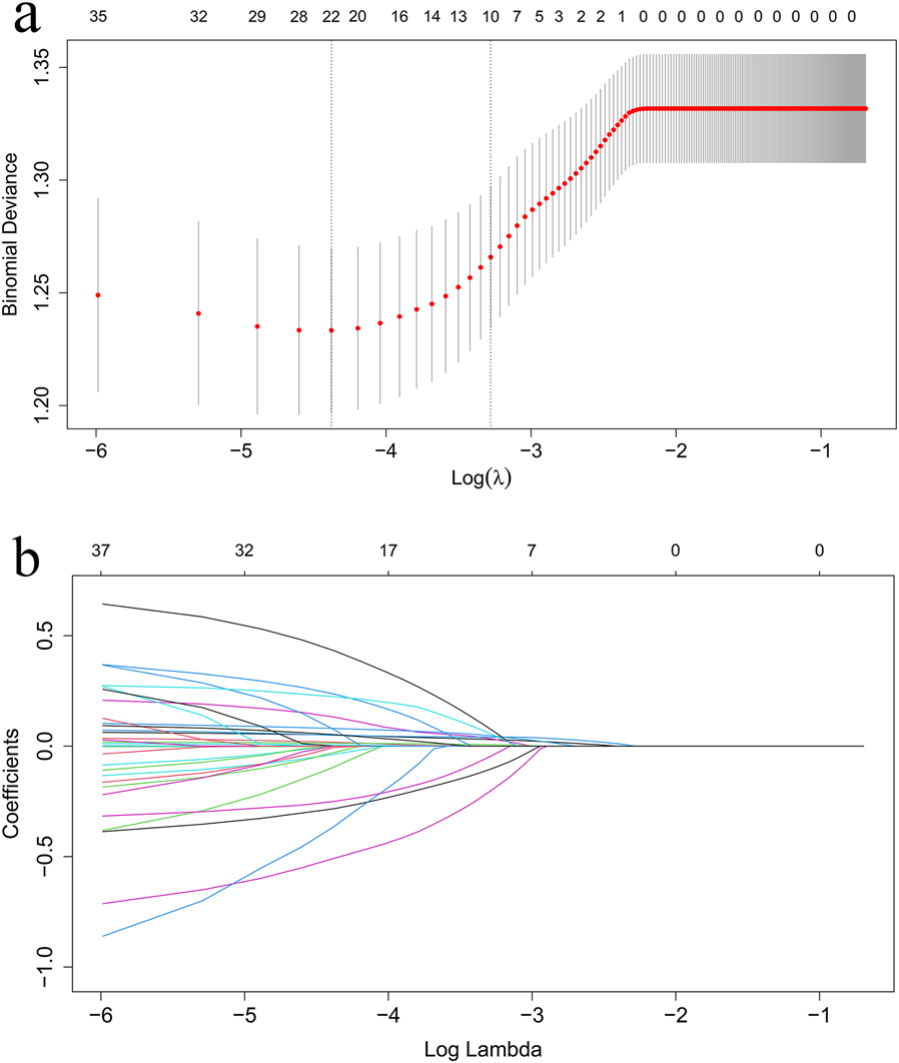
**a** Process of Selection variables by LASSO regression. **b** Process of selecting the parameter of best lambda value by cross-validation

All variables from the baseline characteristics in Table 1 were incorporated into the LASSO regression analysis with tenfold cross-validation, yielding a λ value of 0.038. Eventually, 10 covariates associated with the prognosis were selected, including age, SOFA score, anion gap, serum phosphate, RDW, congestive heart failure, cerebrovascular disease, VF, norepinephrine, and transthoracic echocardiography, as shown in Fig. 3. The Cox regression is shown in Table 3. In model I, without adjusting for any confounding factors, the HRs (95% CI) for 90-day all-cause mortality of the low ACC group (< 8.86) and high ACC group (> 10.32) were 1.188 (0.972–1.453) and 1.673 (1.219–2.296), suggesting that high ACC was a risk factor for 90-day mortality in patients with CA (*P* < 0.05). However, after adjusting for the 10 variables selected through the LASSO analysis, it could be concluded that low ACC or high ACC was an independent risk factor for 90-day all-cause mortality in patients with CA (1.317 1.069–1.621, *P* = 0.010; 1.391 1.005–1.926, *P* = 0.047). In the Cox analysis of the relationship between ACC levels and 360-day all-cause mortality risk in patients with CA, similar results were observed (1.285 1.054–1.567, *P* = 0.013; 1.477 1.086–2.009, *P* = 0.013). The final conclusion drawn was that both low ACC (< 8.86) and high ACC (> 10.32) were independent risk factors for 90-day and 360-day all-cause mortality in patients with CA (*P* < 0.05).

**Table 3:** Cox proportional hazard analysis of all-cause mortality in patients with CA between the three groups

	Variable	Model I			Model II			Model III		
		HR	95% CI	*P*-value	HR	95% CI	*P*-value	HR	95% CI	*P*-value
Original cohort	90-day mortality									
	8.86–10.32	1			1			1		
	< 8.86	1.188	0.972–1.453	0.092	1.279	1.040–1.573	0.020	1.317	1.069–1.621	0.010
	> 10.32	1.673	1.219–2.296	0.001	1.411	1.021–1.949	0.037	1.391	1.005–1.926	0.047
	360-day mortality									
	8.86–10.32	1			1			1		
	< 8.86	1.132	0.935–1.371	0.205	1.248	1.024–1.520	0.028	1.285	1.054–1.567	0.013
	> 10.32	1.733	1.285–2.337	< 0.001	1.488	1.096–2.020	0.011	1.477	1.086–2.009	0.013
Weighted cohort	90-day mortality									
	8.86–10.32	1			1			1		
	< 8.86	1.279	1.025–1.596	0.029	1.249	0.992–1.573	0.059	1.301	1.029–1.645	0.028
	> 10.32	1.589	1.178–2.143	0.002	1.458	1.074–1.979	0.016	1.426	1.025–1.984	0.035
	360-day mortality									
	8.86–10.32	1			1			1		
	< 8.86	1.255	1.015–1.552	0.036	1.226	0.982–1.532	0.073	1.281	1.022–1.604	0.031
	> 10.32	1.595	1.192–2.134	0.002	1.475	1.098–1.982	0.010	1.457	1.054–2.014	0.023

Original cohort:

Model I: No covariate adjustments were made. Model II: Adjusted for age, SOFA score, anion gap, phosphate, and RDW

Model III: Built upon model II, further adjusted for the comorbidities including congestive heart failure, VF, cerebrovascular disease, and the proportion of receiving transthoracic echocardiography and norepinephrine

Matched cohort:

Model I: No covariate adjustments were made

Model II: Adjusted for age, SOFA score, WBC count, hemoglobin, RDW, MCV, AST, BUN, creatinine, anion gap, and phosphate

Model III: Built upon model II, further adjusted for the comorbidities including congestive heart failure, VF, cerebrovascular disease, malignant tumor, and the usage of norepinephrine

To validate the stability of the results, variables with *P* < 0.1 in the weighted cohort in Table 1 were included in another Cox analysis, as shown in Table 3. The results remained stable, indicating that both low ACC (< 8.86) and high ACC (> 10.32) were independent risk factors for 90-day and 360-day all-cause mortality in patients with CA (*P* < 0.05).

In order to further validate the stability of the results, sensitivity analysis was used, revealing that both low ACC (< 8.86) and high ACC group (> 10.32) were independent risk factors for 90-day and 360-day all-cause mortality in patients with CA (*P* < 0.05). These findings were consistent with the previously mentioned results, demonstrating the stability, as shown in Additional file 2: Tables S1–S3.

### Kaplan–Meier survival curve analysis

**Fig. 4:**
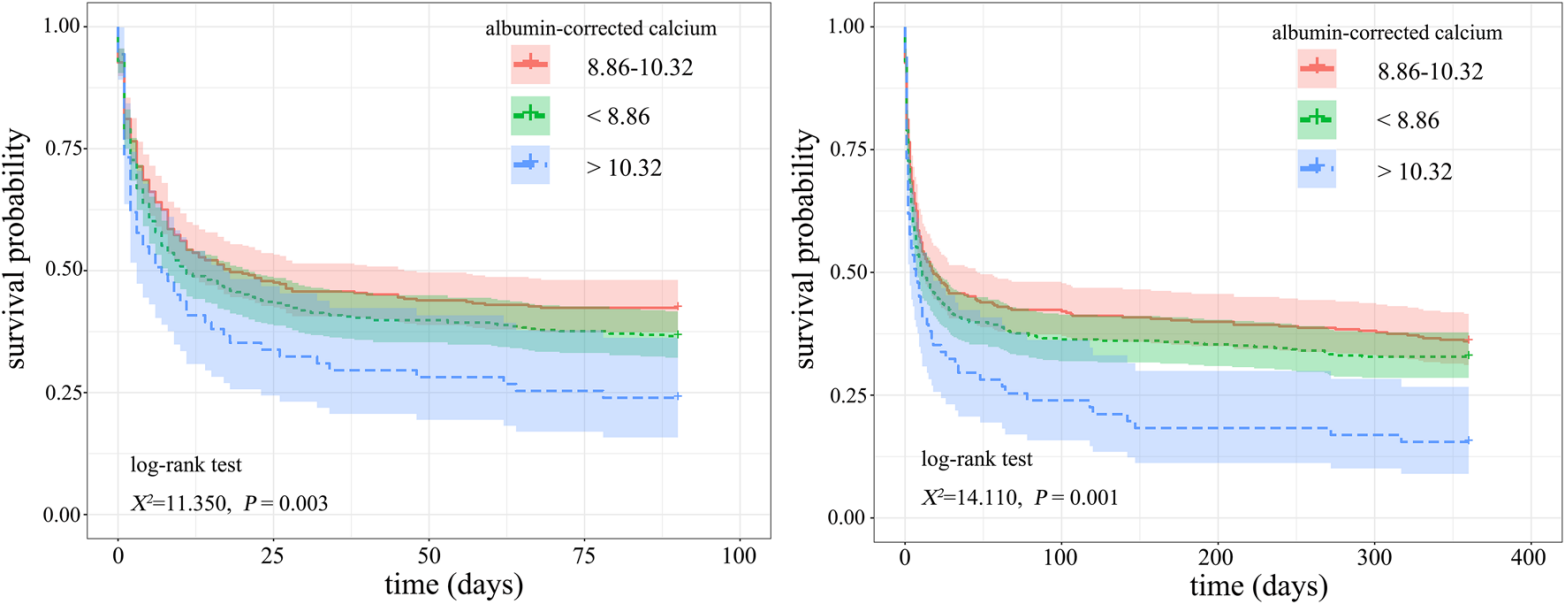
Kaplan–Meier survival curve of 90-day (left) and 360-day (right) cumulative survival rates for different levels of albumin-corrected calcium (ACC)

**Fig. 5:**
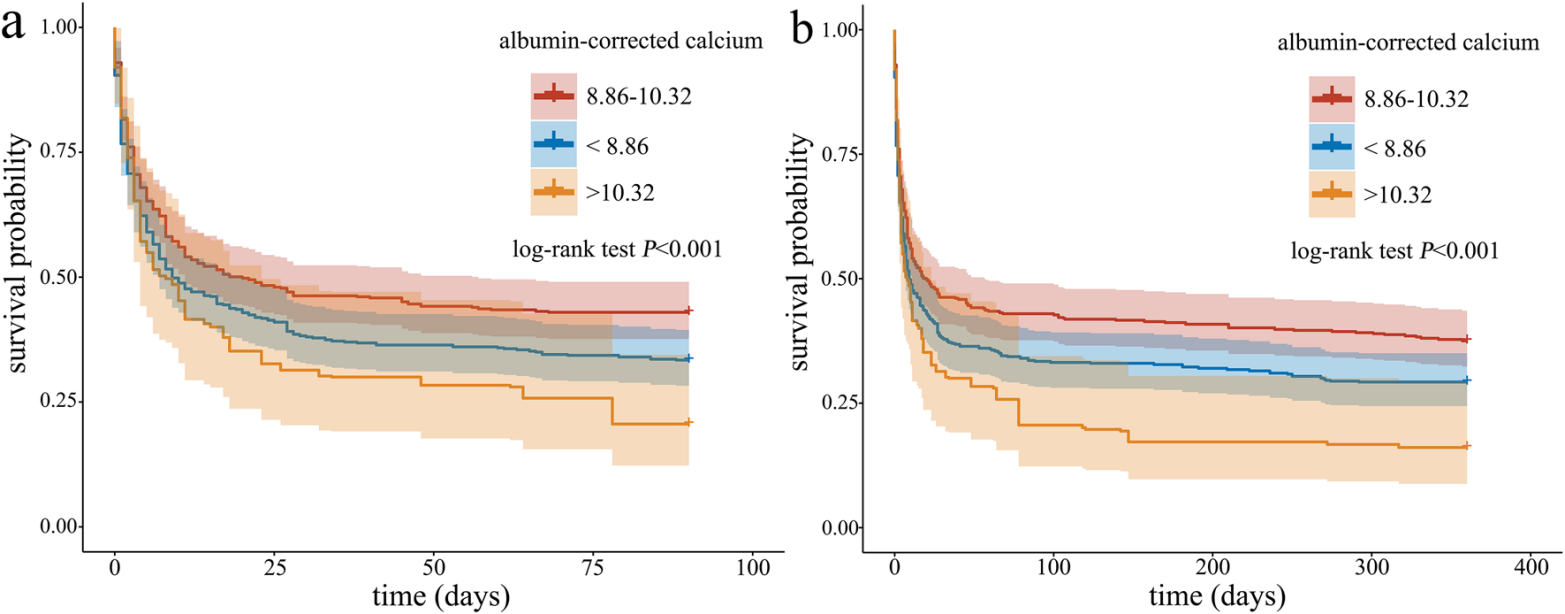
Kaplan–Meier survival curve of 90-day (left) and 360-day (right) cumulative survival rates After IPTW for different levels of albumin-corrected calcium (ACC)

The Kaplan–Meier survival curves, as indicated in Fig. 4, showed that the 90- and 360-day cumulative survival rates of the CA patients in the low ACC (< 8.86) and high ACC (> 10.32) groups were lower than that in the moderate ACC (8.86–10.32) group, and the 90-day and 360-day survival rates were lowest in the high ACC (> 10.32) groups (log-rank test, χ^2^ = 11.350,* P* = 0.003; χ^2^ = 14.110,* P* = 0.001). After IPTW, the results of Kaplan–Meier survival curves remained consistent, as shown in Fig. 5.

## Discussion

In this retrospective study, we obtained the cut-off values for ACC used in risk stratification for patients with CA, which were 8.86 and 10.32, respectively. After adjusting for the 10 variables selected through the LASSO analysis, the Cox regression model suggested that low ACC (< 8.86) or high ACC (> 10.32) was an independent risk factor for 90-day and 360-day all-cause mortality among CA patients (*P* < 0.013). Furthermore, the results of Cox analysis in the weighted cohort remained stable, indicating that both low ACC (< 8.86) and high ACC (> 10.32) were independent risk factors for 90-day and 360-day all-cause mortality in patients with CA (*P* < 0.05). The stability of the results was further validated by the sensitivity analysis. The Kaplan–Meier survival curves before and after IPTW analyses suggested that 90-day and 360-day cumulative survival rates of the patients with CA in the low ACC (< 8.86) and high ACC (> 10.32) groups were lower than that in the moderate ACC (8.86–10.32) group (*P* < 0.05). This facilitated risk stratification for patients with CA, providing a theoretical basis for clinical physicians to identify high-risk patients.

A significant proportion of the high mortality is attributed to electrolyte disturbances, including potassium, sodium, and calcium among ICU patients. Although abnormal serum calcium concentrations are not factored into the acute physiology and chronic health evaluation prediction system for assessing prognosis in ICU patients, they are highly prevalent in this population. Disruptions in calcium metabolism have the potential to influence the excitability of the neuromuscular system, resulting in arrhythmias, multi-organ dysfunction, and in severe cases, coma, and CA [13]. The relationship between different serum calcium levels and mortality of various diseases was explored by previous studies. Shiyovich et al. in 2018 found that serum calcium (< 9.12 mg/dL and > 9.86 mg/dL) was an independent predictor of in-hospital mortality among AMI patients and a U-shaped association was noted [10]. Additionally, Yang and colleagues acknowledged that hypocalcemia was independently linked to midterm and long-term mortality in patients with acute pulmonary thromboembolism [11]. Meanwhile, researchers found that ionized levels of calcium were associated with the severity of sepsis and could independently predict an adverse outcome in very low birth weight infants with sepsis [14]. Low preoperative serum calcium levels were linked to advanced stage tumors. Decreased serum calcium may be related to the progression of esophageal cancer [15]. In a multicenter retrospective cohort study conducted in the United States, which included patients undergoing peritoneal dialysis or hemodialysis, it was observed that hypercalcemia was associated with a mortality risk increase of up to 60% [16]. In addition, calcium was also associated with many other diseases, such as dengue infection [17] and acute coronary syndrome [18], and osteoporotic vertebral compression fractures [19]. However, total calcium was influenced by the body's PH and serum albumin levels, making it less accurate, and the measurement of ionized calcium was more complex and influenced by multiple factors. Therefore, clinicians commonly use ACC to assess ionized calcium levels.

ACC gained the recognition among scholars for its aforementioned advantages; however, research on this topic remained limited. One research indicated a significant association between higher levels of ACC and reduced survival in colorectal cancer patients [20]. Furthermore, some scholars found that elevated serum ACC levels after mechanical thrombectomy were independently linked to adverse outcomes in stroke patients [21]. Additionally, ACC could serve as a predictor for the severity of acute pancreatitis [22]. A recent study conducted by Qin showed an association between ACC and an increased risk of mortality in patients admitted to ICU [9]. However, there was no research on ACC in patients with CA at the time. Thus, we conducted a retrospective analysis including 798 patients diagnosed with CA in the MIMIC database, and a non-linear 'U'-shaped association was observed between ACC and the risk of 90-day and 360-day mortalities. Furthermore, after adjusting for potential confounding factors, ACC abnormalities emerged as independent risk factors for both 90-day and 360-day mortalities. The conclusion reached in our study was essentially consistent with that obtained by Qin and colleagues. In contrast to other studies, our study conducted two types of Cox regression analyses using variables selected from different methods, and the results remained stable. What is more, sensitivity analysis further validated the stability of the results. These findings can provide clinicians with valuable insights.

This study has several strengths. Firstly, it was the first to investigate the correlation between ACC and the prognosis of patients with CA. Additionally, this study provided cut-off values (8.86 and 10.32) for risk stratification in patients with CA. Furthermore, the stability of the results was verified from different perspectives. What is more, this study utilized the IPTW method to alleviate or adjust for potential selection bias and confounding factors and subsequently employed Cox and survival analyses for further investigation. However, there were also certain limitations. It was a retrospective study. At the same time, information regarding the usage of calcium supplements, vitamin D receptor activators, and other medications potentially influencing calcium metabolism were not available, which could potentially impact the conclusions. Therefore, further research was needed in the future.

## Conclusion

Both low ACC (< 8.86) and high ACC (> 10.32) were independent risk factors for 90-day and 360-day all-cause mortality in patients with CA (*P* < 0.05). It may provide a theoretical basis for clinical physicians to identify high-risk patients, and further research is needed to confirm it in the future.

### Supplementary Information

**Additional file 1.** STROBE statement—checklist of items to include in cohort study reports.

**Additional file 2: Table S1.** Sensitivity analysis between the three groups excluding patients who received albumin transfusions in the three days prior to admission. **Table S2.** Sensitivity analysis between the three groups excluding patients with malignant tumor. **Table S3.** Sensitivity analysis between the three groups excluding patients with cirrhosis.

## Data Availability

The datasets utilized in this study were accessible within the MIMIC-IV database (https://physionet.org/content/mimiciv/2.0/).

## Abbreviations

ACC: Albumin-corrected calcium
AKI: Acute kidney injury
ALT: Alanine aminotransferase
AMI: Acute myocardial infarction
APACHE: Acute physiology and chronic health evaluation
AST: Aspartate aminotransferase
BUN: Blood urea nitrogen
CA: Cardiac arrest
CI: Confidence intervals
DVT: Deep vein thrombosis
HR: Hazard ratio
IABP: Intra-aortic balloon pump
ICU: Intensive Care Unit
LASSO: Least absolute shrinkage and selection operator
LOS: Length of stay
MCV: Mean corpuscular volume
MIMIC-IV: Medical information mart for intensive care IV
MV: Mechanical ventilation
RCS: Restricted cubic spline
RDW: Red blood cell distribution width
SOFA: Sequential organ failure assessment
VF: Ventricular fibrillation
WBC: White blood cell
