# Supplementary material for: The association between albumin-corrected calcium and prognosis in patients with cardiac arrest: a retrospective study based on the MIMIC-IV database
Source: Eur J Med Res. 2024 Apr 24;29:251. doi: 10.1186/s40001-024-01841-4 (PMC11044335; doi:10.1186/s40001-024-01841-4)
Supplement: Supplementary file 2 — Additional file 2: Table S1. Sensitivity analysis between the three groups excluding patients who received albumin transfusions in the three days prior to admission. Table S2. Sensitivity analysis between the three groups excluding patients with malignant tumor. Table S3. Sensitivity analysis between the three groups excluding patients with cirrhosis. [file 40001_2024_1841_MOESM2_ESM.docx]

**Table S1** Sensitivity analysis between the three groups excluding patients who received albumin transfusions in the three days prior to admission

| Variable | Model I | | |  | Model II | | |  | Model III | | |
| --- | --- | --- | --- | --- | --- | --- | --- | --- | --- | --- | --- |
|  | HR | 95%CI | *P*-value |  | HR | 95%CI | *P*-value |  | HR | 95%CI | *P*-value |
| **90-day mortality** |  |  |  |  |  |  |  |  |  |  |  |
| 8.86-10.32 (n=328) | 1 |  |  |  | 1 |  |  |  | 1 |  |  |
| ＜8.86 (n=399) | 1.204 | 0.982-1.477 | 0.074 |  | 1.296 | 1.050-1.599 | 0.016 |  | 1.335 | 1.081-1.648 | 0.007 |
| ＞10.32 ( n=71) | 1.722 | 1.250-2.373 | 0.001 |  | 1.458 | 1.051-2.022 | 0.025 |  | 1.446 | 1.034-1.995 | 0.031 |
| **360-day mortality** |  |  |  |  |  |  |  |  |  |  |  |
| 8.86-10.32 (n=328) | 1 |  |  |  | 1 |  |  |  | 1 |  |  |
| ＜8.86 (n=399) | 1.144 | 0.942-1.388 | 0.175 |  | 1.261 | 1.032-1.540 | 0.023 |  | 1.300 | 1.064-1.589 | 0.010 |
| ＞10.32 ( n=71) | 1.788 | 1.321-2.420 | <0.001 |  | 1.543 | 1.133-2.102 | 0.006 |  | 1.530 | 1.122-2.087 | 0.007 |

Model I: No covariates were adjusted.

Model II: adjusted for age, SOFA score, anion gap, phosphate and RDW.

Model III: based on model II, adjusted for the comorbidities including congestive heart failure, VF, cerebrovascular disease, the proportion receiving transthoracic echocardiography and norepinephrine.

Note: Variable set A, the variables adjusted in Model III.

**Table S2** Sensitivity analysis between the three groups excluding patients with malignant tumor

| Variable | Model I | | |  | Model II | | |  | Model III | | |
| --- | --- | --- | --- | --- | --- | --- | --- | --- | --- | --- | --- |
|  | HR | 95%CI | *P*-value |  | HR | 95%CI | *P*-value |  | HR | 95%CI | *P*-value |
| **90-day mortality** |  |  |  |  |  |  |  |  |  |  |  |
| 8.86-10.32 (n=328) | 1 |  |  |  | 1 |  |  |  | 1 |  |  |
| ＜8.86 (n=399) | 1.253 | 1.006-1.561 | 0.044 |  | 1.361 | 1.085-1.707 | 0.008 |  | 1.398 | 1.114-1.754 | 0.004 |
| ＞10.32 ( n=71) | 1.693 | 1.203-2.383 | 0.003 |  | 1.476 | 1.044-2.086 | 0.028 |  | 1.529 | 1.080-2.167 | 0.017 |
| **360-day mortality** |  |  |  |  |  |  |  |  |  |  |  |
| 8.86-10.32 (n=328) | 1 |  |  |  | 1 |  |  |  | 1 |  |  |
| ＜8.86 (n=399) | 1.215 | 0.985-1.498 | 0.070 |  | 1.350 | 1.087-1.676 | 0.007 |  | 1.389 | 1.118-1.726 | 0.003 |
| ＞10.32 ( n=71) | 1.799 | 1.304-2.481 | <0.001 |  | 1.585 | 1.143-2.196 | 0.006 |  | 1.644 | 1.184-2.284 | 0.003 |

Model I: No covariates were adjusted.

Model II: adjusted for age, SOFA score, anion gap, phosphate and RDW.

Model III: based on model II, adjusted for the comorbidities including congestive heart failure, VF, cerebrovascular disease, the proportion receiving transthoracic echocardiography and norepinephrine.

Note: Variable set A, the variables adjusted in Model III.

**Table S3** Sensitivity analysis between the three groups excluding patients with cirrhosis

| Variable | Model I | | |  | Model II | | |  | Model III | | |
| --- | --- | --- | --- | --- | --- | --- | --- | --- | --- | --- | --- |
|  | HR | 95%CI | *P*-value |  | HR | 95%CI | *P*-value |  | HR | 95%CI | *P*-value |
| **90-day mortality** |  |  |  |  |  |  |  |  |  |  |  |
| 8.86-10.32 (n=328) | 1 |  |  |  | 1 |  |  |  | 1 |  |  |
| ＜8.86 (n=399) | 1.264 | 1.026-1.557 | 0.028 |  | 1.371 | 1.104-1.703 | 0.004 |  | 1.403 | 1.129-1.744 | 0.002 |
| ＞10.32 ( n=71) | 1.800 | 1.282-2.528 | 0.001 |  | 1.538 | 1.087-2.176 | 0.015 |  | 1.523 | 1.075-2.159 | 0.018 |
| **360-day mortality** |  |  |  |  |  |  |  |  |  |  |  |
| 8.86-10.32 (n=328) | 1 |  |  |  | 1 |  |  |  | 1 |  |  |
| ＜8.86 (n=399) | 1.192 | 0.977-1.455 | 0.083 |  | 1.324 | 1.085-1.641 | 0.006 |  | 1.368 | 1.112-1.683 | 0.003 |
| ＞10.32 ( n=71) | 1.809 | 1.309-2.501 | <0.001 |  | 1.588 | 1.140-2.211 | 0.006 |  | 1.572 | 1.127-2.193 | 0.008 |

Model I: No covariates were adjusted.

Model II: adjusted for age, SOFA score, anion gap, phosphate and RDW.

Model III: based on model II, adjusted for the comorbidities including congestive heart failure, VF, cerebrovascular disease, the proportion receiving transthoracic echocardiography and norepinephrine.

Note: Variable set A, the variables adjusted in Model III.
